# Bladder Neck Obstruction: Experience and Management in a Sperm Bank

**DOI:** 10.3390/life13030842

**Published:** 2023-03-20

**Authors:** Maria Neve Hirsch, Francesco Pallotti, Fabiana Faja, Alessandra Buonacquisto, Gaia Cicolani, Anna Chiara Conflitti, Silvia Di Chiano, Andrea Lenzi, Francesco Lombardo, Donatella Paoli

**Affiliations:** Laboratory of Seminology–Sperm Bank “Loredana Gandini”, Department of Experimental Medicine, “Sapienza” University of Rome, 00161 Rome, Italy

**Keywords:** primary bladder neck obstruction, transurethral incision, retrograde ejaculation, sperm cryopreservation

## Abstract

Primary Bladder Neck Obstruction (PBNO) management provides medical and surgical treatment, such as transurethral incisions that can lead to retrograde ejaculation. The aim of this study was to investigate the maintenance of anterograde ejaculation and semen quality before and after this surgical procedure. A retrospective evaluation was carried out between 2011 and 2020. A total of 73 patients diagnosed with PBNO were recruited. Ejaculatory function, semen quality, and the fertility of recruited subjects were evaluated. Semen parameters—Baseline, 8.2% of patients were oligozoospermic and 12.3% had a semen volume below the WHO 2010 fifth percentile. Post-surgery, 20% of patients were oligozoospermic. We detected a significant decrease in total sperm number, a significant increase in the number of abnormal forms, and a reduction in the leukocyte concentration. Ejaculatory function—A total of 7.7% of patients reported anejaculation after transurethral incision of the bladder neck. Fertility—9.2% of the patients already had children before surgery; 13.8% had naturally conceived children in the years following surgery; 76.9% had no desire for paternity at the time. Our data have important implications for sperm bank management. The alterations in semen parameters and the risk of anejaculation suggest that the use of sperm cryopreservation before surgery for PBNO should be encouraged.

## 1. Introduction

Primary Bladder Neck Obstruction (PBNO) is a condition where the bladder neck fails to open sufficiently during voiding, in absence of other anatomic obstructions, resulting in the obstruction of the urinary flow and/or increased sphincter activity [1]. There are still uncertainties regarding PBNO’s etiology, incidence and prevalence in men, in view of the fact that available epidemiological data were collected from too-specific populations. Kaplan et al. [2] reported a 54% incidence of PBNO in a retrospective study of 137 men younger than 50 years old, with chronic voiding dysfunction. Nitti et al. [3] found a 47% incidence, in a prospective study of 85 males between the ages of 18 and 45 years, of lower urinary tract symptoms. Moreover, the spectrum of PBNO manifestations includes voiding, obstructive, storage and irritative symptoms, alone or in combination, which may be commonly confused with many different conditions (chronic abacterial prostatitis, neurogenic bladder dysfunction, pelvic pain, psychogenic voiding dysfunction) [3]. PBNO management may vary following observation, ranging from continued follow-up to medical and surgical treatment. Medical treatment with an alpha-adrenergic antagonist is the first-line treatment, but it is unusual for young men to continue on this treatment with time, and only 30% of patients use this medication in the long-term [4]. The transurethral incision of the bladder neck is an effective treatment of PBNO. The incision can be either unilateral or bilateral and are normally performed at the 5 or 7 o’clock positions or, in some cases, in the 2 and 10 o’clock positions. Despite its effectiveness, the main concern with this procedure is the development of retrograde ejaculation, which may theoretically occur even after a minor unilateral incision. Retrograde ejaculation rate after transurethral incision of the bladder neck of patients undergoing bilateral incision has been reported in different studies, varying from 0% to 100% (0% to 16% when performing a unilateral incision) [5,6,7,8,9,10,11,12,13,14,15,16] (Supplementary Table S1) Despite clinical PBNO resolution, the patient may experience an important surgical side-effect that may impact not only male fertility, but also sexual satisfaction.

For this reason, preoperative semen cryopreservation, especially in patients interested in maintaining fertility, should be recommended [17]; in the event of retrograde ejaculation, the availability of cryopreserved semen samples prevents any future need of sperm retrieval from either urine samples or testicular surgery. In the latter case, the theoretical risk of subsequent testicular damage is avoided, as well as its costs and any preoperative anxiety [18,19,20]. The specialist in reproductive medicine, working in a multi-disciplinary team, should recommend access to a fertility preservation service when-ever a patient faces a condition or treatment that might interfere either with spermatogenesis and genome integrity or ejaculation mechanisms [21]. Unfortunately, only few studies have focused on this aspect. The aim of this study was to investigate the preservation of anterograde ejaculation and the semen quality pre- and post-bladder neck incision, in a retrospectively recruited caseload of patients who referred were to our Sperm Bank for pre-surgical semen cryopreservation.

## 2. Materials and Methods

### 2.1. Patients

The retrospective evaluation of data was approved by the Ethics Committee “Sapienza”—Policlinico Umberto I (Prot. 182/11). Written informed consent was obtained from all study participants.

We retrospectively recruited patients attending the Laboratory of Seminology- Sperm Bank “Loredana Gandini” (Department of Experimental Medicine, Policlinico Umberto I—“Sapienza” University of Rome) for semen cryopreservation, prior to the surgical treatment of primary bladder neck obstruction, between 2011 and 2020. In particular, 65 patients, aged 36.0 ± 9.0 years, underwent yearly follow-up andrological visits and, during follow-up, 25 patients agreed to provide a semen sample after a median of 12 months following surgery (6–36 months). Transurethral incision of the bladder neck was performed on all the subjects. Exclusion criteria were the use of gonadotoxic drugs in the two years prior to cryopreservation, any abdominal/genital surgical treatment other than bladder neck transurethral incision, and coexisting benign prostatic hyperplasia. All patients who successfully cryopreserved their semen were included in a yearly clinical andrological follow-up. Data regarding medical history (including surgical treatment, previous diseases, drug treatments, smoking habits, andrological diseases, fertility, ejaculatory function) were collected either from medical records or personal interview.

### 2.2. Semen Analysis

Semen samples were collected by masturbation after 2–7 days abstinence. All samples were allowed to liquefy at 37 °C for 60 min, and were assessed according to WHO (2010). The following variables were taken into consideration: volume (mL), sperm concentration (×10^6^/mL), total sperm number (n × 10^6^ per ejaculate), progressive motility (%), and morphology (% abnormal forms). In particular, sperm concentration was assessed using a Makler chamber. Sperm motility was analyzed as soon as possible, when fluidification was completed, and it was classified as progressive and non-progressive (WHO, 2010). Sperm morphology was evaluated, both on a fresh preparation and on stained smears, by May–Grünwald–Giemsa staining. We evaluated the percentage of abnormal forms and we described the more frequent forms. For sperm motility and morphology evaluation, at least 200 spermatozoa were examined.

### 2.3. Statistical Analysis

Continuous variables are presented as means, medians and standard deviations. Differences between groups were evaluated by the Wilcoxon Matched-Pairs Signed Ranks Test or the paired Student’s *t*-test, based on the data distribution as evaluated by the Kolmogorov–Smirnov test. Categorical variables are presented as counts and percentages and were compared by the Fisher’s Exact test. The probability values are 2-sided and a *p*-value < 0.05 was considered to be statistically significant. All computations were carried out with Statistical Package for the Social Sciences (SPSS) 25.0 (SPSS Inc., Chicago, IL, USA).

## 3. Results

### 3.1. Semen Quality before Surgery

Between 2011 and 2020, a total of 73 PBNO patients (median age of 37.2) provided at least one semen sample for semen cryopreservation before surgery (T0) (only four patients were requested to collect two or more samples, due to severely reduced semen volume and/or quality). Table 1 provides the age and semen parameters of patients before treatment. Only six patients (8.2%) were oligozoospermic, whereas nine patients (12.3%) had a semen volume below the fifth percentile (WHO 2010).

### 3.2. Semen Quality after Surgery

We analyzed semen semples provided by 25 patients, an average of 12 months following surgery (6–36 months). We detected a significant decrease in total sperm number and an increase in the number abnormal forms following surgery, while leukocyte concentrations in semen samples were reduced, as shown in Table 2. The mainly abnormal forms were amorphous heads, small heads, bent necks and coiled tails. It is noteworthy that the mean values of all semen parameters were above the corresponding fifth percentile (WHO 2010). Overall, post-surgery oligozoospermic patients represented 20% of the total. We also observed that, post-surgery, 36% of patients had a semen volume below the fifth percentile, as outlined in the WHO 2010 guidelines; nonetheless, the mean reduction of semen volume did not reach statistical significance.

### 3.3. Ejaculatory Function

All the patients were interviewed regarding their ejaculatory function. Excluding eight patients lost to follow up, only 5 out of the remaining 65 patients reported anejaculation after transurethral incision of the bladder neck (7.7%). The average age of the patients who lost anterograde ejaculation was 37.4 ± 6.9 years, which was comparable to the age of those patients who preserved ejaculation. Five more subjects reported a relapse of the bladder neck obstruction, but none of them had anejaculation. Only one subject performed a second semen cryopreservation before re-surgery, but, after 78 months, he did not report any alterations in the ejaculatory function.

### 3.4. Fertility

**Figure 1:**
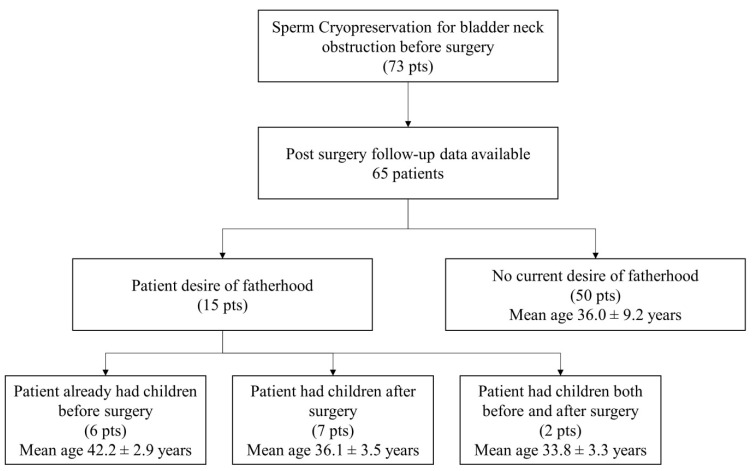
Flowchart of the fertility investigation of the subjects.

We collected follow-up data regarding the fertility of 65 patients. Six patients (9.2%) already had children before surgery, while nine (13.8%) patients had naturally conceived children in the years following surgery. These latter patients achieved pregnancy in a median of 30 months (mean ± SD: 32.6 ± 16.6 months). At the moment of the interview, fifty patients (76.9%) reported no children and no desire for paternity, but, until now, none of our patients have requested cryopreserved semen for its use in assisted fertilization techniques following surgery (Figure 1).

## 4. Discussion

**Figure 2:**
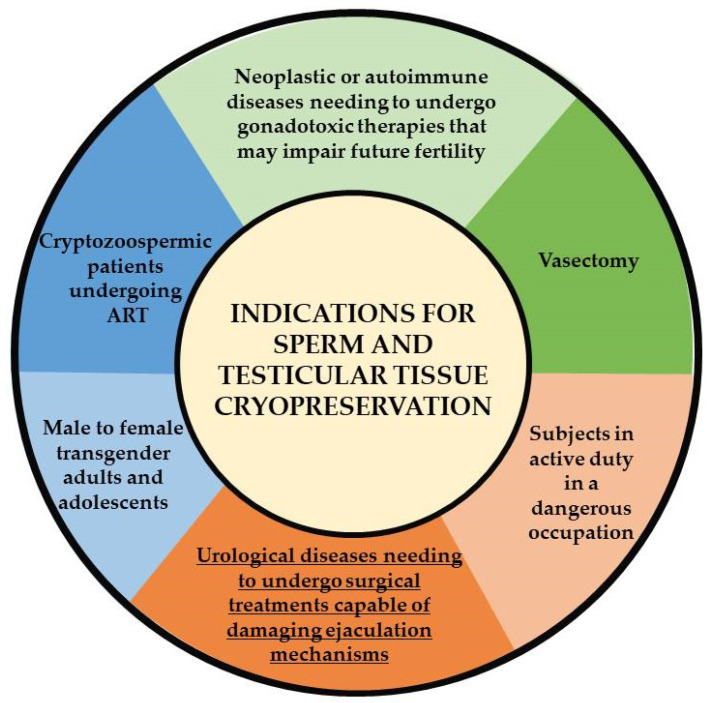
Indications for sperm and testicular tissue cryopreservation.

PBNO frequently causes voiding dysfunction in young men, and the most effective treatment is bladder neck incision, which can be associated with retrograde ejaculation, compromising the chance of fatherhood in young patients. In fact, treatment may impact both male infertility and also sexual satisfaction. For this reason, semen cryopreservation before surgery should be routinely recommended. Cryopreservation is a technique that can be used to keep spermatozoa alive indefinitely and, therefore, it acquires extreme importance in the setting of patients undergoing a surgical treatment that could impair the subjects’ fertility [22]. Primary indications to undergo sperm cryopreservation are mainly cancer treatments, but also include 145 autoimmune and urological diseases (Figure 2).

In this study we evaluated the semen parameters and preservation of anterograde ejaculation before and after surgical treatment of PBNO, in patients who were referred to our Sperm Bank for semen cryopreservation. In our caseload, all patients underwent a transurethral incision of the bladder neck. The majority of patients who were referred to the Sperm Bank underwent transurethral incision of the bladder neck, whereas a small proportion of patients underwent Transurethral Resection of the Prostate (TURP). These patients were excluded from the study, because there was a component of benign prostatic hyperplasia.

Regarding the semen parameters, only two studies evaluated the sperm count after bladder neck incision. One was performed by Hedlund et al., who analyzed semen samples of 16 patients before and after surgery [11]. The incision was unilateral for seven patients and bilateral for nine subjects. These authors did not find a change in semen volume, sperm concentration or fructose levels in seminal plasma pre- and post-surgery. It should be noted that the authors evaluated only two semen parameters: semen volume and sperm concentration. On the other hand, Kochakarn and Lertsithichai detected a decrease in sperm concentration 6 to 12 months after surgery (a 70% mean decrease from preoperative values) in 35 men aged 36–46 years. Additionally, in this study, the authors only considered the sperm concentration when evaluating the fertility of the patients [4].

To date, literature data on the effect of surgery on this benign condition are scant, referring to small caseloads. This represents a limit for the reproductive health specialists in this field, as we have little evidence related to these fertility issues that may be useful, both for the patients and the sperm bank management. Most literature studies evaluate fertility counseling among men of reproductive age who are receiving potentially gonadotoxic chemotherapy [23]. For this reason, we conducted a study on 73 PBNO patients who cryopreserved spermatozoa before bladder neck surgery from 2011- to 2020. We reported the experience of our sperm bank, which has been operating since 1996, and has assisted around 6500 patients. Although most patients are referred to our bank for fertility preservation procedures before antineoplastic therapy, each year, several patients cryopreserve their semen before urological procedures, which may compromise fertility. In particular, since 1996, the number of patients affected by PBNO is 214.

In our caseload, we detected a significant reduction in total sperm number and an increase in the percentage of abnormal forms, although 80% of patients showed post-surgical semen parameters that resulted in values above the WHO 2010 fifth percentile for up to 36 months, and 20% were oligozoospermic.

Although no testicular damage is expected from PBNO surgery, we hypothesize that the reduction in the total sperm number can be explained by a sub-obstruction of the ejaculatory ducts that occurs after surgery, due to a lower incision or the growth of scars. In these patients, a transrectal ultrasound study may be a reasonable way to verify the integrity of the ejaculatory ducts. Alternatively, it is possible that, at least in some patients, the total sperm number decrease could be related to a “partial” post-surgical retrograde ejaculation, with a reduced quantity of semen volume flowing anterogradely. We observed a trend reduction in post-surgery sperm concentration and, in 36% of patients, a semen volume reduction was observed. In this subset of patients, it may be possible to investigate the presence of retrograde ejaculation through a search for spermatozoa in post-ejaculation urine samples. Unfortunately, in our caseload, it was possible to do this test only in one patient, with negative results.

Different studies have reported that retrograde ejaculation is less likely to occur when performing a unilateral incision (0% to 16% reported rate) [5,6,9,11,16,24,25]. In our caseload, we observed a prevalence of post-surgery anejaculaton of 7.7%. The incision is usually stopped proximally to the verumontanum. Since ejaculatory ducts open distally to the verumontanum, after surgery, the closure of the proximal urethra is maintained by the contraction of undamaged smooth urethral musculature, at the level of the verumontanum [11]. While this should guarantee the preservation of ejaculation in most patients, a number of subjects develops retrograde ejaculation, but the reasons are still unclear.

Finally, to our knowledge, this is the first study that uses the available fertility data of patients following neck bladder incision. In our caseload, around 14% of patients had naturally conceived children in the years following surgery, and none of our patients had used cryopreserved semen.

## 5. Conclusions

Results from this study have relevant implications for PBNO patients’ management. First, our data highlight the need of semen cryopreservation prior to surgery for PBNO, given the non-negligible risk of retrograde ejaculation and reduced total sperm number after surgery. Since there is no clinical predictor of anejaculation or semen quality after surgery, a cryopreserved semen sample allows patients to access assisted fertilization techniques, without resorting to surgical testicular sperm extraction, which is invasive and expensive, could induce psychological pressure, and may induce testicular damage in some patients. Second, it appears important to perform semen quality evaluations in these patients during follow up, allowing for a more informed counseling and management of the cryopreserved sample, with further implications for the sperm bank’s management of the sample. Obviously, it is imperative to maintain the cryopreserved sample for patients who lose ejaculation after surgery, or in patients who present with altered semen parameters during follow up, but it may be possible to discard those good-quality samples after a careful clinical evaluation. This recommendation is also made in light of our data on natural fertility after surgery. It is nonetheless recommended to discuss fertility preservation with all patients of reproductive age who will receive treatment that carries a possible risk of iatrogenic infertility. It is mandatory that the andrologist, with other relevant specialists (seminologist, infectious diseases specialist, psychologist, gynecologist, etc.), assist the patients with both malignant and non-malignant diseases, discussing possible fertility preservation options and offering counselling and a careful follow up of testicular function, aiming at evaluating semen quality, hormone profile, as well as using testicular ultrasound.

## Figures and Tables

**Table 1:** T Summary of baseline (before surgery) semen parameters of the whole caseload. Continuous variables are presented as mean ± SD and median (in brackets).

	Age (Years)	Semen Volume (mL)	Sperm Concentration (×10^6^/mL)	Total Sperm Number (×10^6^)	Progressive Motility (%)	Abnormal Forms (%)	Leukocytes (×10^6^)
T0 (73 pts)	36.0 ± 9.0 (37.2)	3.3 ± 1.8 (3.0)	101.3 ± 105.5 (77.0)	335.8 ± 509.3 (215.6)	43.3 ± 15.3 (50.0)	83.5 ± 9.0 (83.0)	1.0 ± 1.4 (0.6)

**Table 2:** Baseline (T0) vs. follow-up (T1) sperm parameters. T1 controls were analyzed a median of 12 months following surgery. Continuous variables are presented as mean ± SD and median (in brackets).

	Semen Volume (mL)	Sperm Concentration (×10^6^/mL)	Total Sperm Number (×10^6^)	Progressive Motility (%)	Abnormal Forms (%)	Leukocytes (×10^6^)
T0 (25 pts)	2.9 ± 1.7 (2.5)	107.5 ± 118.8 (94.0)	239.3 ± 206.4 (180.0)	45.2 ± 15.0 (50.0)	82.0 ± 10.7 (83.0)	0.8 ± 0.7 (0.6)
T1 (25 pts)	2.4 ± 1.7 (2.0)	72.6 ± 59.3 (78.0)	145.0 ± 127.5 (116.0)	43.6 ± 18.7 (55.0)	84.6 ± 8.7 (87.0)	0.5 ± 0.3 (0.4)
*p* value	0.159	0.058	0.015	0.806	0.049	0.031

## Data Availability

The data underlying this article will be shared on reasonable request to the corresponding author.

## Supplementary Materials

The following supporting information can be downloaded at: https://www.mdpi.com/article/10.3390/life13030842/s1, Table S1: Data from the literature on retrograde ejaculation rate after bladder neck incision.
