# Supplementary material for: Bladder Neck Obstruction: Experience and Management in a Sperm Bank
Source: Life (Basel). 2023 Mar 20;13(3):842. doi: 10.3390/life13030842 (PMC10059771; doi:10.3390/life13030842)
Supplement: Supplementary file 1 [file life-13-00842-s001.zip › life-2234785-supplementary.pdf]

Supplementary Table S1. Data from the literature on retrograde ejaculation rate after bladder neck incision.

Reference	Year	Enrolled pts	Incision	Retrograde ejaculation (%)
Andersen et al. [12]	1980	28	Bilateral	0
Webster et al. [6]	1980	16	Unilateral	0
Moisey et al. [15]	1982	38	Unilateral	16
Delaere et al. [13]	1983	32	Bilateral	36
Hedlund et al. [11]	1985	61	Uni/Bilateral	5/5
Christensen et al. [14]	1985	131	Bilateral	22
Norlen et al. [9]	1986	23	Uni/bilateral	0/100
Mobb et al. [16]	1988	64	Unilateral	16
Kaplan et al. [5]	1994	31	Unilateral	0
El-Baz et al. [10]	1995	62	Bilateral	8
Trockman et al. [7]	1996	36	Bilateral	27
Neykov et al. [8]	1998	35	Bilateral	8.5
Kochakarn et al. [4]	2003	35	Unilateral	0
Yang et al. [24]	2008	32	Unilateral	0
